# Supplementary material for: Can smart policies solve the sand mining problem?
Source: PLoS One. 2021 Apr 2;16(4):e0248882. doi: 10.1371/journal.pone.0248882 (PMC8018655; doi:10.1371/journal.pone.0248882)
Supplement: S1 Appendix — (PDF) [file pone.0248882.s001.pdf]

Figures & Appendix

Can Smart Policies Solve the Sand Mining Problem?

Michael Hübler[*], Frank Pothen[†]

November 16, 2020

Abstract

Part I provides the figures with the main simulation results as well as supplementary figures showing descriptive statistics, a robustness check and a sensitivity analysis as supplementary online materials. Part II provides the supplementary online appendix. The online appendix first describes, in a nontechnical way, the model setup in terms of represented regions and sectors as well as functional forms. Based on that information, the appendix describes the data sources of the numerical model calibration. It explains the representation and disaggregation of the sand sector and the definition of the related policies. It then discusses the limitations of the model analysis. The final section contains the mathematical formulation of the model.

[*]Corresponding author. Email: michael.huebler@agrar.uni-giessen.de, Institute for Agricultural Policy and Market Research, Center for International Development and Environmental Research (ZEU), Justus Liebig University Giessen, phone: +49-641-99-37052, fax: +49-641 99-37059, Senckenbergstr. 3, 35390 Gießen, Germany; Institute for Environmental Economics and World Trade, Leibniz University Hannover, Germany.

[†]Email: frank.pothen@imw.fraunhofer.de, Fraunhofer Center for International Management and Knowledge Economy IMW, Neumarkt 9, 04109 Leipzig, Germany.

I Figures

1 Main simulation results

The following figures show the main simulation results. Figure 1 depicts the sand tax rate as a function of the reduction of total sand extraction in all sand extracting (exporting) Southeast Asian countries. These graphs can be interpreted as marginal reduction (abatement) cost curves. Figures 2 and 3 show countries' relative welfare changes vs. the reduction of total sand extraction.

Figure 1
Marginal reduction (abatement) cost curves

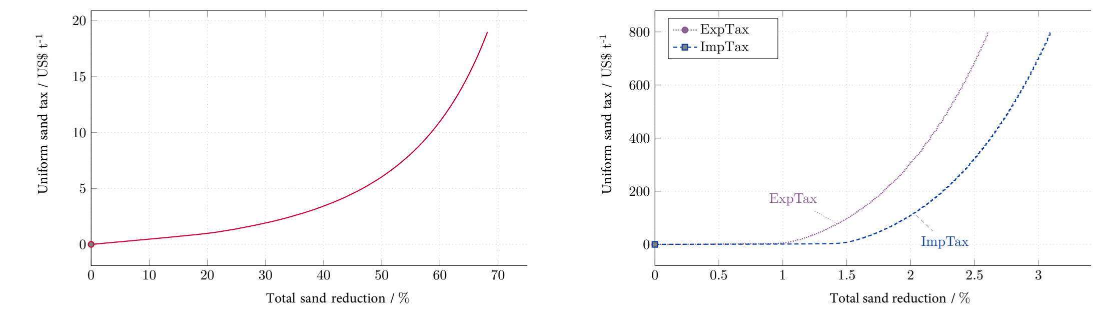

(a) Output tax (OutTax) (b) Export (ExpTax) & import tax (ImpTax)

Figure 2
Regional welfare effects

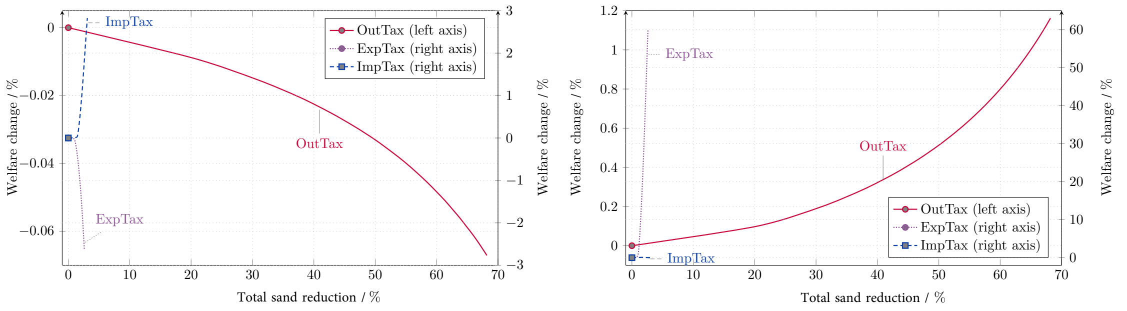

(a) Singapore (SGP) (b) Cambodia (KHM)

Figure 1 is described in the main text. Figures 2 and 3 depict country-specific relative welfare effects (where gains are positive) as a function of the corresponding reduction of total sand extraction. The major exporters can achieve welfare gains by imposing a uniform export tax on sand in all exporting countries (ExpTax), whereas the minor exporters lose. With a total sand extraction reduction of 2.6%, the largest exporter KHM achieves the maximum welfare gain of 60%, and MMR achieves ca. 1.4% gain compared

Figure 3
Regional welfare effects

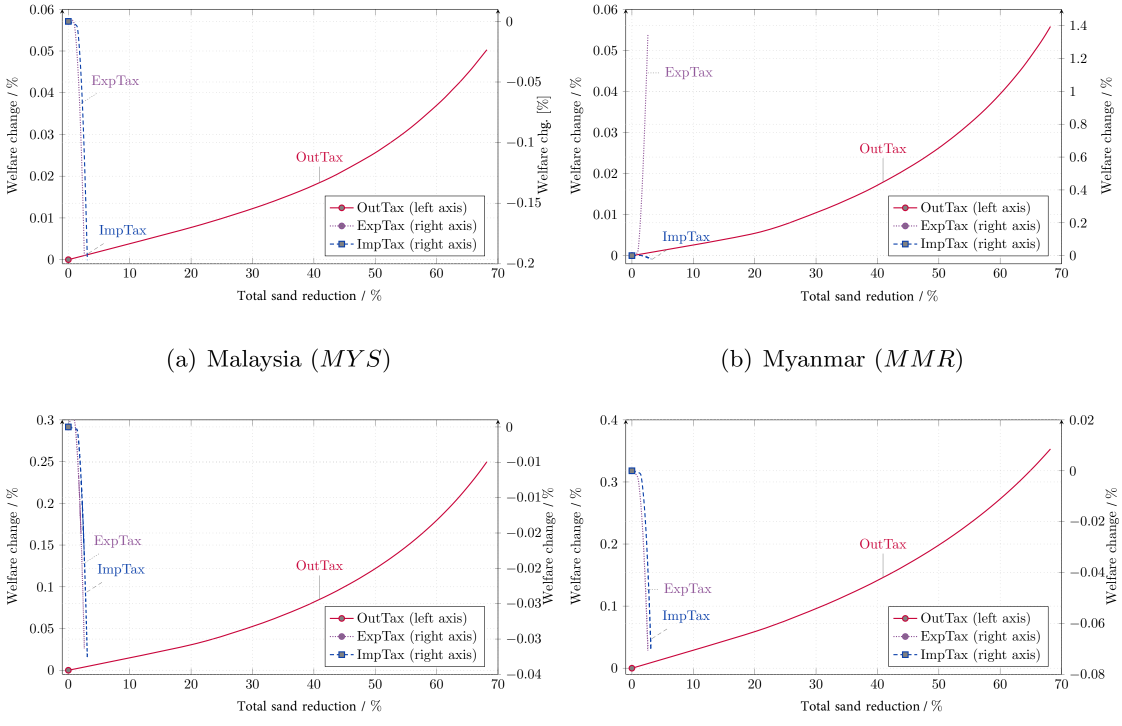

(a) Malaysia (MYS)

(b) Myanmar (MMR)

(c) Philippines (PHL)

(d) Vietnam (VNM)

to the benchmark scenario of having no sand policy, notably, at an unrealistic tax rate of approximately US-$800 per ton. If the export tax is replaced by the Singaporean import tax (ImpTax), all exporters will become worse off than without any sand policy because the tax revenues accrue to SGP. The welfare effects of the import tax are, however, small (far below 1%). SGP, on the contrary, gains almost 3% from the import tax but loses almost 3% due to the export tax, compared to the benchmark scenario of no sand policy.

The use of the output tax in all exporting countries (OutTax) results in a positive, convex and increasing welfare effect as a function of the corresponding sand reduction for all exporters. Whereas the total sand extraction can be reduced by approximately 70%, the achievable welfare gains vary from 0.06% in MYS and MMR to over 0.3% in PHL and VNM, and up to 1.4% in KHM. SGP's corresponding welfare effect mirrors those of the exporters: the welfare effect as a function of the sand reduction is negative, concave and decreasing; the magnitude of the welfare loss reaches 0.08

2 Descriptive statistics

Figure D1 displays the distribution of Singapore's (SGP's) sand and gravel imports (data classifications H1–H3) by source countries based on import values for recent years as published in the United Nations Comtrade database in 2015/16 (UN Comtrade, 2016). In accordance with these data, the subsequent policy analysis will concentrate on the largest sand suppliers: Cambodia (KHM), Malaysia (MYS), Myanmar (MMR), the Philippines (PHL) and Vietnam (VNM). Note that in the model's benchmark year 2011, Vietnam's sand exports to Singapore were small. Because Indonesia's sand exports were minor in all years, Indonesia is left out of the analysis.

Figure D1
Distribution of Singapore's sand imports

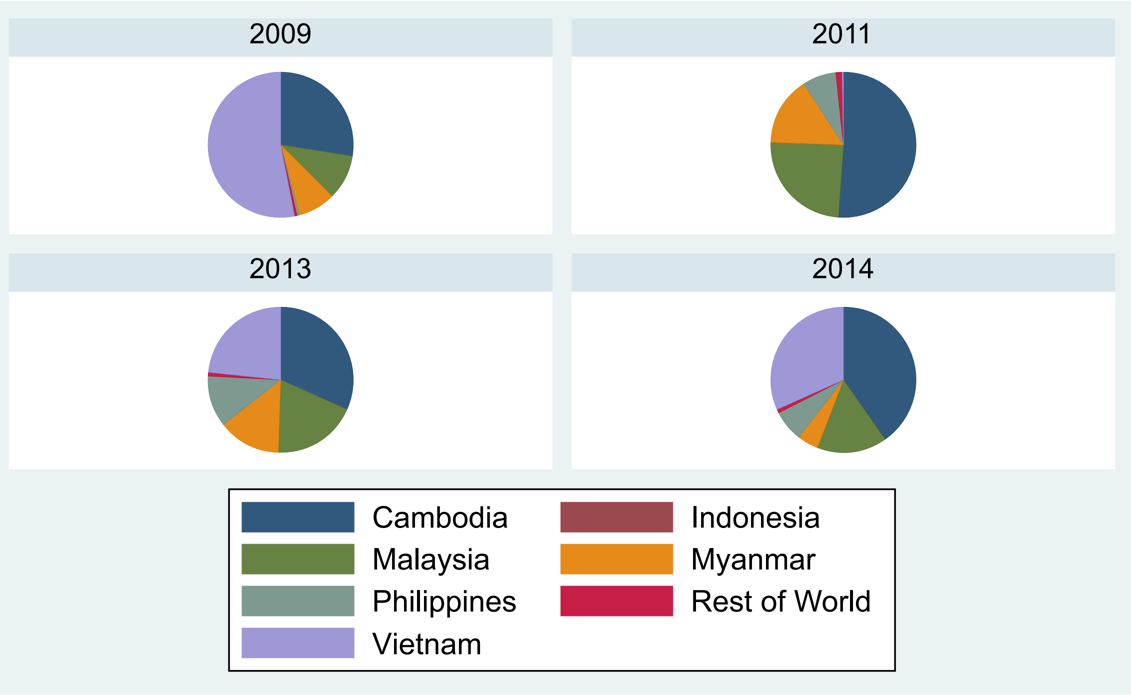

By source countries (UN Comtrade, 2016)

3 Robustness check

According to the variation in the UN Comtrade (2016) data over time and considering Singapore's land reclamation plans, the actually traded and used sand volumes vary by a factor of 3.5 and can be expected to increase by up to a factor of seven in the future. Hence, the alternative robustness check scenario HigDem will take this variation into

account by imposing the policy instruments on an economy, in which Singapore's sand demand is assumed to rise fivefold as a realistic medium value.

Figure R1
Marginal reduction (abatement) cost curves with extended sand demand

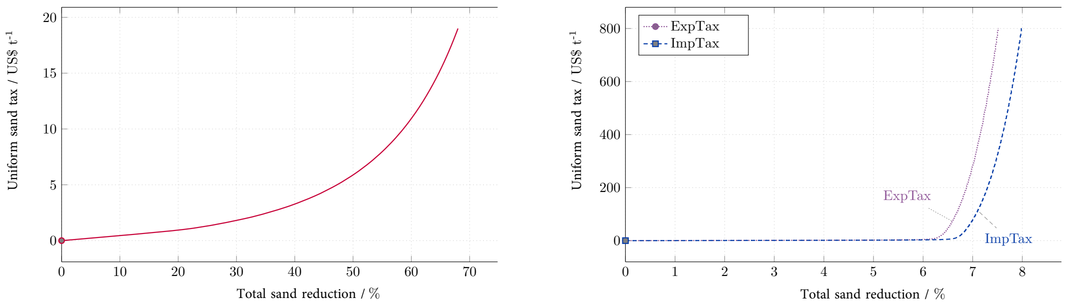

(a) Output tax (OutTax/HigDem) (b) Exp. (ExpTax/HigDem) & imp. tax (ImpTax)

Figures R1 to R3 illustrate the simulation results for the HigDem scenario with the assumption that *SGP*'s sand demand will increase fivefold compared to the previous standard policy scenarios. Figure R1 depicts the sand tax rate as a function of the reduction of total sand extraction in all sand-extracting (exporting) Southeast Asian countries. The graphs can be interpreted as marginal reduction (abatement) cost curves. Figures R2 and R3 show countries' relative welfare changes vs. the reduction of total sand extraction.

Figure R2
Regional welfare effects with extended sand demand

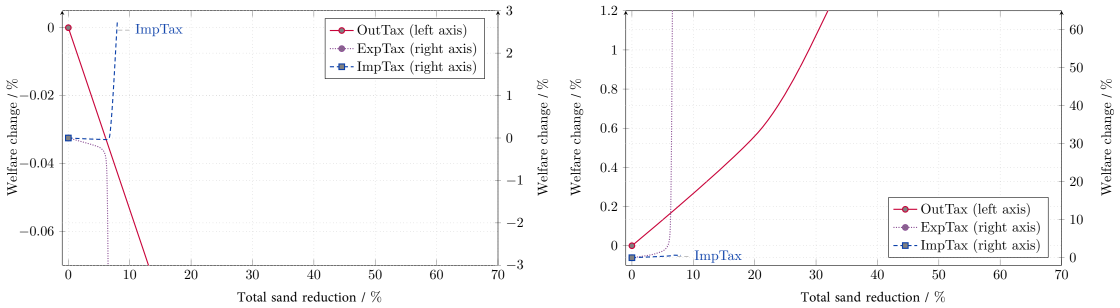

(a) Singapore (*SGP*/HigDem) (b) Cambodia (*KHM*/HigDem)

According to this robustness check, the welfare effects of the export tax (ExpTax/HigDem) that are negative for the importer *SGP* but positive for the exporters, rise by almost an order of magnitude (except in *PHL*) compared to the standard scenario (ExpTax). The corresponding maximum achievable total sand reduction more than doubles to 7.5% under ExpTax/HigDem and 8.0% under ImpTax/HigDem (figure R1 (b)). The welfare gain that *SGP* can achieve via the import tax (ImpTax/HigDem) remains

Figure R3
Regional welfare effects with extended sand demand

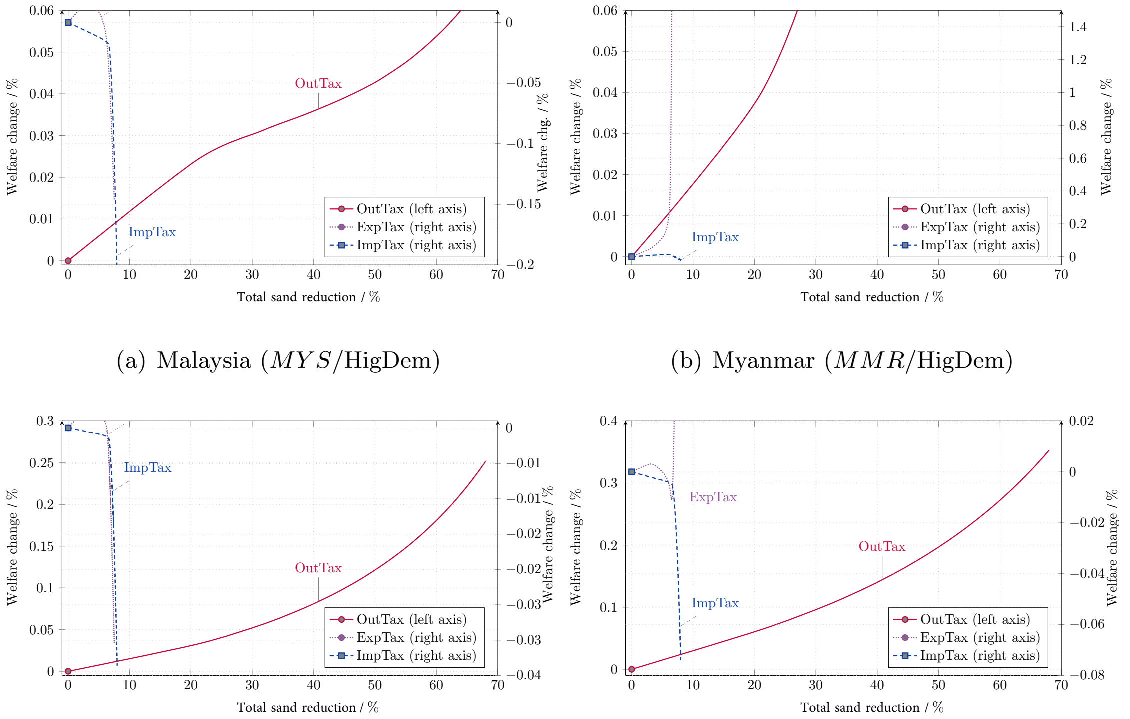

(a) Malaysia (MYS/HigDem)

(b) Myanmar (MMR/HigDem)

(c) Philippines (PHL/HigDem)

(d) Vietnam (VNM/HigDem)

the same as before (3% under ImpTax). Most welfare effects and the corresponding total sand reductions induced by the output tax (OutTax/HigDem), in contrast, hardly change compared to the respective values under the standard scenarios, except in SGP and MMR, where welfare effects rise by almost an order of magnitude compared to the effects under the standard scenario (OutTax).

4 Sensitivity analysis

To assess the uncertainty in key model parameter values, we perform a sensitivity analysis.

First, we vary the trade elasticity parameter values θ_i of all sectors i by $\pm$ one standard deviation. The means (as reported in table A4) and standard deviations are taken from Caliendo and Parro (2015). The remaining standard deviations corresponding to the means taken from Eaton and Kortum (2002) are set to one.

Second, the construction ($CONS$) and non-metallic minerals ($NMMS$) sectors absorb most of the total sand supply. Hence, we change the standard value of $\sigma^Z = 0$, i.e., the elasticity of substitution between intermediate inputs including sand (as

illustrated in figure A2 and reported in table A3), in these two sectors to 0.25 and 0.75, respectively.

Figure S1
Margin. abatem. cost curves with variation in trade and input elasticities

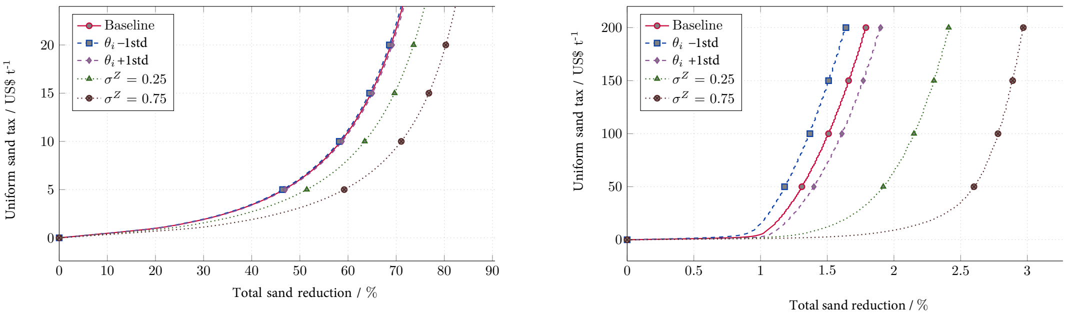

(a) Output tax (OutTax)

(b) Export tax (ExpTax)

Figure S2
Regional welfare effects with variation in trade and input elasticities

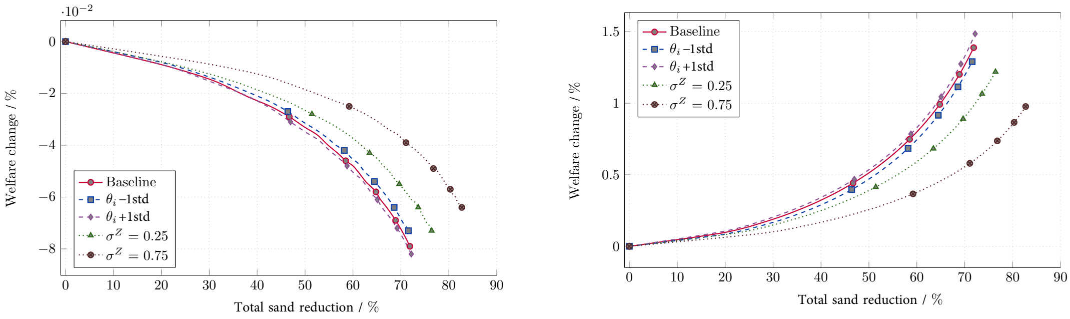

(a) Output tax (OutTax), Singapore (SGP)

(b) Output tax (OutTax), Cambodia (KHM)

Figures S1 to S3 show the simulation results of the sensitivity analysis. According to the results, the effect on sand extraction and welfare is significant. For instance, the achievable sand reduction via the export tax doubles, and SGP's welfare loss created by the output tax decreases by 50% if 0.75 is assumed. Although these effects are dominated by the uncertainty of the future sand demand discussed above, alterations of elasticities can increase or decrease the differential between the effects of trade policy and output taxation.

Figure S3
Regional welfare effects with variation in trade and input elasticities

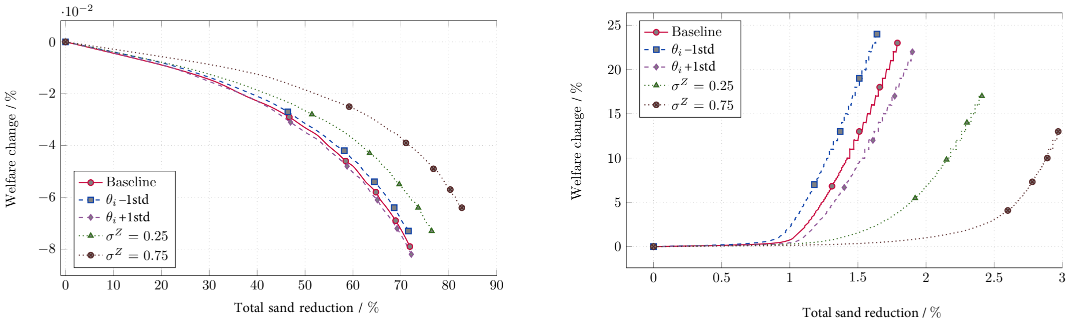

(a) Export tax (OutTax), Singapore (SGP) (b) Export tax (ExpTax), Cambodia (KHM)

II Appendix

1 Basic model structure

1.1 Regions

Table A1 lists 16 model regions r (alternatively, s). The column $SAND$ indicates whether there is a sand extracting sector in r.

Table A1
Regions in the model

r	Region	$SAND$	r	Region	$SAND$
KHM	Cambodia	Yes	KOR	Korea, Republic of	No
MYS	Malaysia	Yes	TWN	Taiwan	No
MMR	Myanmar	Yes	IDN	Indonesia	No
PHL	Philippines	Yes	USA	United States	No
VNM	Vietnam	Yes	EUR	Europe (EFTA)	No
SGP	Singapore	No	JPN	Japan	No
THA	Thailand	No	ROA	Rest of Asia	No
CHN	China	No	ROW	Rest of the World	No

1.2 Sectors

Table A2 presents 16 model sectors (goods) i (alternatively, j).

Table A2
Sectors in the model

i	Sector
$AGRI$	Agriculture
$COAL$	Coal
$CRUD$	Crude oil
$NGAS$	Natural gas
$PETR$	Refined petroleum
$FOOD$	Food production
$SAND$	Sand and gravel
$OTMN$	Other mining excl. sand
$MANU$	Manufacturing
$NMMS$	Non-metallic minerals
$EINS$	Energy-intensive sectors
$ELEC$	Electricity
$TRNS$	Transport
$CONS$	Construction
$SERV$	Services
$(INVS$	Investment)

The investment good sector $INVS$ provides a nontradable good and is hence excluded from the analysis of international trade. It is not used as an intermediate good input either. All other goods can either be used for final consumption or as intermediate inputs in production. Section 5.5 will additionally introduce international (global) transport services, which are required for shipping goods but are not treated as a normal production sector.

2 Nested CES functions

2.1 Consumption

In each model region s, a representative consumer maximizes her utility U_s by choosing the optimal consumption bundle of all composite goods. She has nested constant elasticity of substitution (CES) preferences over sectoral composites. The preference structure is depicted by figure A1. The nested preferences allow for a differentiated degree of substitutability between individual goods in different nests. σ denotes the elasticity of substitution between goods in each nest.

At the top level, the function combines a bundle of energy goods C_s^E with a non-energy bundle C_s^N. The elasticity of substitution between them is denoted by σ^C. The consumption of energy goods coal ($COAL$), crude oil ($CRUD$), gas ($NGAS$), refined petroleum ($PETR$) and electricity ($ELEC$) is aggregated in the energy bundle C_s^E. The elasticity of substitution between energy goods is σ^{CE}. C_s^N is the corresponding bundle

Figure A1
Nesting structure of the consumption (utility) function

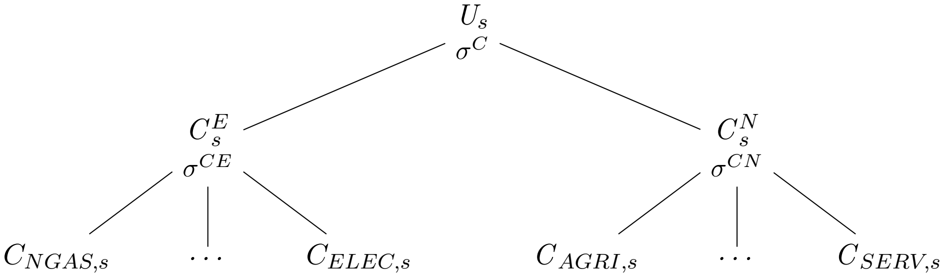

of non-energy goods combined with the elasticity σ^{CN}.

2.2 Production

In each sector i of each region r, representative producers provide a continuum of differentiated varieties of the sector's good. They use the primary factors of labor $L_{i,r}$ and capital $K_{i,r}$ as well as intermediate inputs from all sectors as inputs. The producers minimize their input costs subject to the production function (technology) depicted in figure A2 by choosing the cost-minimizing input bundle $\tilde{q}_{i,r}(z_{i,r})$. Whereas most Eaton and Kortum (2002) type models assume that factors and intermediate inputs are combined in a Cobb-Douglas fashion, the underlying model follows Pothen and Hübler (2018) by implementing a nested CES production structure.

Figure A2
Nesting structure of the production function

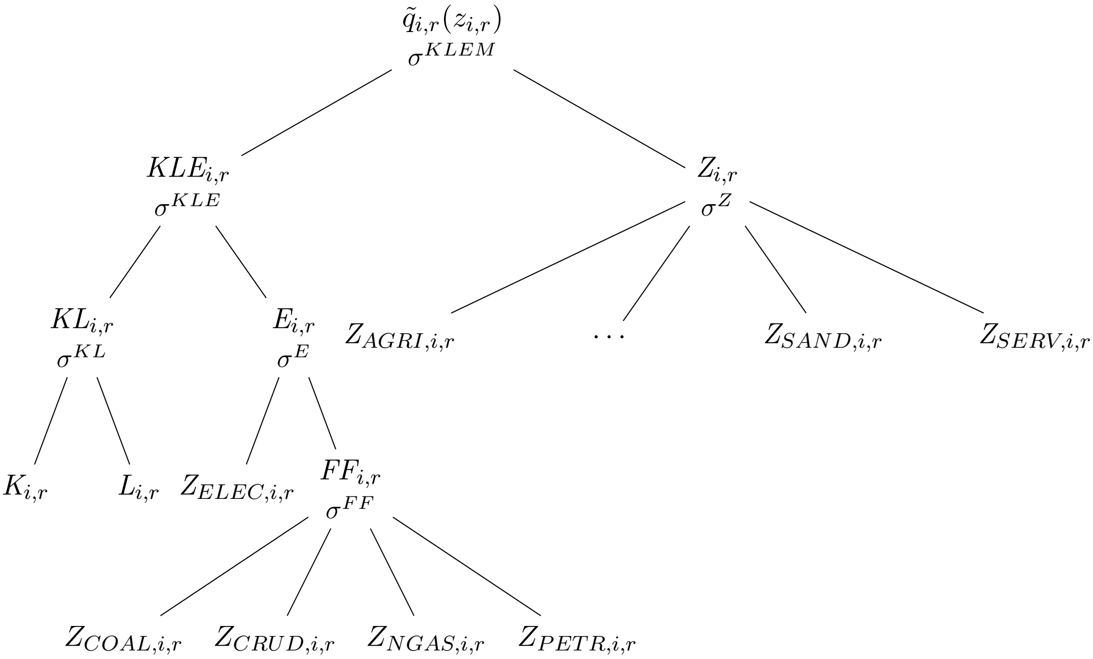

Inputs of labor $L_{i,r}$ and capital $K_{i,r}$ are combined in the nest $KL_{i,r}$ assuming an elasticity of substitution $\sigma^{KL} = 1$ (Cobb-Douglas) between them. The fossil fuels nest $FF_{i,r}$ combines inputs of coal ($Z_{COAL,i,r}$), crude oil ($Z_{CRUD,i,r}$), natural gas ($Z_{NGAS,i,r}$) and refined petroleum ($Z_{PETR,i,r}$). The corresponding elasticity of substitution is denoted by σ^{FF}. The energy nest $E_{i,r}$ combines fossil fuel inputs with electricity inputs with the elasticity of substitution σ^{E}. This assumption reflects the idea that electricity serves a different purpose in production processes than that of fossil fuels. The inputs of energy and value added are combined in the $KLE_{i,r}$ nest with the corresponding elasticity of substitution σ^{KLE}. This structure is consistent with van der Werf (2008) who has shown that substitution between energy and value added matches the empirical data well. The aggregate $KLE_{i,r}$ is combined with non-energy intermediate inputs with the elasticity of substitution σ^{KLEM} to obtain the cost-minimal input bundle $\tilde{q}_{i,r}(z_{i,r})$. Individual non-energy inputs are aggregated in the nest $Z_{i,r}$ with the elasticity of substitution $\sigma^{Z} = 0$ (Leontief). Intermediate inputs of $SAND$ are also included in the non-energy input nest $Z_{i,r}$. Accordingly, the demand for $SAND$ does not react elastically to changes in prices because sand is (currently) an indispensable input in construction.

3 Model calibration

3.1 Input-output data

The Global Trade Analysis Project (GTAP) dataset, version 9 (Aguiar et al., 2016) is the main data source providing input-output data for the benchmark year 2011. The data cover consumption, production and international trade as well as policy parameters such as subsidies and taxes. These data are used to calibrate the input value shares[1] and corresponding outputs of the CES functions described in section 2.

Data on sand and gravel trade are taken from UN Comtrade (2016). We use two six-digit harmonized system (HS) items to quantify the flows of sand: HS 250590 (sands; natural, other than silica and quartz sands, whether or not colored, other than metal-bearing sands of chapter 26) and HS 2517 (pebbles, gravel, crushed stone for concrete aggregates for road or railway ballast, shingle or flint; macadam of slag, dross, etc., tarred granules, chippings, powder of stones of heading No. 2515 and 2516).[2] The physical

[1]A larger input value share implies larger economic effects of changing this input.

[2]For MMR, we use data for 2010 because there are no physical export data available for 2011. For VNM and MYS, we use the average of the other three regions as physical exports are unavailable (VNM) or the implied prices are implausibly high (MYS).

extraction of sand and gravel is taken from the materialflows.net database (Lutter et al., 2015).

3.2 CES elasticities

The CES functions characterized in section 2 require the choice of elasticities of substitution, which are not covered by input-output datasets. Because the design of our model has been inspired by the established MIT EPPA (Emissions Prediction and Policy Analysis) model (Paltsev et al., 2005), we draw on the elasticities of substitution used there.

Table A3 presents the used values of the elasticity of substitution σ. They are assumed to be equal across all sectors and regions. A larger elasticity implies better substitutability between the attached inputs and hence more flexibility in terms of adjustments to policy changes. As a consequence, negative welfare effects of taxation will likely become smaller.

Table A3
CES elasticities of substitution

	Elasticity of substitution between	Value
Consumption:		
σ^{CE}	Energy goods	0.40
σ^{CN}	Non-energy goods	0.25
σ^{C}	Energy and non-energy aggregates	0.25
Production:		
σ^{KLEM}	KLE and intermediates	1.50
σ^{KLE}	Value added and energy	0.40
σ^{Z}	Non-energy intermediates	0.00
σ^{KL}	Capital and labor	1.00
σ^{E}	Electricity and fossil fuels	0.50
σ^{FF}	Fossil fuels	1.00
σ^{Q}	Varieties	2.00

σ = elasticity of substitution; values are taken from Paltsev et al. (2005).

3.3 Trade elasticities

Table A4
Sectoral trade elasticities

i	Sector	θ_i	Source
$AGRI$	Agriculture	9.11	CP, agriculture
$COAL$	Coal	13.53	CP, mining
$CRUD$	Crude oil	13.53	CP, mining
$NGAS$	Natural gas	13.53	CP, mining
$PETR$	Refined petroleum	64.85	CP, petroleum
$FOOD$	Food production	2.62	CP, food
$SAND$	Sand and gravel	13.53	CP, mining
$OTMN$	Other mining excl. sand	13.53	CP, mining
$NMMS$	Non-metallic minerals	2.41	CP, minerals
$EINS$	Energy-intensive sectors	3.13	CP, chemicals
$ELEC$	Electricity	12.91	CP, electrical
$MANU$	Manufacturing	8.28	EK
$TRNS$	Transport	8.28	EK
$CONS$	Construction	8.28	EK
$SERV$	Services	8.28	EK

CP = Caliendo and Parro (2015, p. 18, 99% sample) with the corresponding sector's name in CP; EK = Eaton and Kortum (2002); i = sector; θ_i = EK parameter governing the trade elasticity.

Table A4 displays the values of the sector-specific shape parameter of the Fréchet distribution θ_i required for the Eaton and Kortum trade model. Whenever possible, the values are based on Caliendo and Parro (2015). In sectors, for which no estimate is available, we use the value of 8.28 according to Eaton and Kortum (2002). A larger value of θ_i reflects a narrower distribution and hence less variation in productivities (Pothen and Hübler, 2018) resulting in less flexibility in terms of adjustments to policy changes. As a consequence, the possible gains from trade via Ricardian specialization in varieties (Eaton and Kortum, 2002) will decrease, and policy impacts on trade will likely become stronger.

4 The sand sector

4.1 Functional form

The design of the sand sector ($SAND$) follows the CES production function (technology) utilized by the other sectors and illustrated in figure A2.

4.2 Disaggregation

The GTAP 9 database (Aguiar et al., 2016) contains an other mining (OMN) sector, which encompasses the extraction of metals and non-metallic minerals. This subsection

describes how the OMN sector is decomposed into a sand and gravel sector ($SAND$) and the remaining other mining sector ($OTMN$). We perform this decomposition for six countries in South-East Asia, i.e., Singapore (SGP) and the five countries that supply the vast majority of Singapore's sand and gravel imports: Cambodia (KHM), Myanmar (MMR), Malaysia (MYS), the Philippines (PHL) and Vietnam (VNM).

Data from two sources are used to decompose the OMN sector. First, imports and exports in both physical and monetary terms are obtained from the UN Comtrade (2016) database. Second, the physical extraction of sand and gravel is obtained from the materialflows.net database (Lutter et al., 2015).

We use the UN Comtrade data for monetary flows of $SAND$ between countries. The domestic use of $SAND$ (i.e., the amount of $SAND$ used in region r that has been produced by region r itself) is not recorded in the UN Comtrade data. Hence, we estimate the amount by subtracting all physical exports of $SAND$ from r's extraction and then multiplying this quantity by the price of $SAND$. This price is approximated by comparing the monetary and physical $SAND$ exports to Singapore. Adding up the purchases of domestically produced and imported $SAND$ yields the total purchase of $SAND$ in each region. As a result, we obtain the following monetary input table for $SAND$, where $SAND$ use is reported in each column, while $SAND$ supply is reported in each row.

Table A5
Sand trade between model regions (in billions of 2011-US$)

	SGP	KHM	MMR	MYS	PHL	VNM
SGP	0.000	0.000	0.000	0.000	0.000	0.000
KHM	0.091	0.008	0.000	0.000	0.000	0.000
MMR	0.027	0.000	0.000	0.000	0.000	0.000
MYS	0.037	0.000	0.000	0.536	0.000	0.000
PHL	0.014	0.000	0.000	0.002	0.367	0.000
VNM	0.001	0.000	0.000	0.001	0.001	1.430

Using these data on $SAND$ flows, we can decompose the OMN sector in the six countries. First, we split the demand for OMN in each region into demand for $SAND$ and $OTMN$ by making the following assumptions:

1. Demand for OMN by the construction sector ($CONS$) is allocated to $SAND$.

2. Demand for OMN by the non-metallic mineral sector ($NMMS$) is assumed to be 50% $SAND$ and 50% $OTMN$.

3. Final demand and investment are assumed to use only $SAND$ but not $OTMN$.

4. Remaining $SAND$ demand is allocated to the remaining sectors proportionally to the sectors' share in total production.

Next, we decompose the trade flows of OMN. In some cases, particularly those of $SAND$ exports from KHM, PHL, and MMR to Singapore, the flows of OMN between countries recorded in the Comtrade data exceed the $SAND$ flows in GTAP. Therefore, we increase the GTAP trade flows to match those in Comtrade. To ensure that the accounting identities remain satisfied, we increase the outputs of the OMN sectors in the supplying countries as well as the corresponding labor inputs. Correspondingly, we need to increase the supply of international transport margins because the additional trade requires transport services. We assume that the additional services are provided by the USA.[3] Except the 17% tax on sand in Vietnam, there are no export tariffs on $SAND$ in 2011 (see OECD, 2018). We obtain import tariffs as well as taxes on OMN for $SAND$ and $OTMN$. Note that Singapore does not levy import tariffs on OMN and thus $SAND$.

Finally, we disaggregate the trade flows (including domestic supply) of OMN into flows of $SAND$ and $OTMN$, ensuring that there is no negative domestic supply of $OTMN$. If necessary, we reduce the demand for $OTMN$ by lowering investments $INVS$. Thereafter, we are able to compute the gross output of $SAND$ and $OTMN$. The inputs into production of OMN are split into inputs in $SAND$ and $OTMN$ according to their shares in the gross output of OMN. Adjusting the income and current account deficits in all regions concludes the disaggregation of the OMN sector.

4.3 Future demand

In the alternative robustness check scenario HigDem, we multiply the sand demand in the year 2011 by a factor of five. This choice is due to two considerations. First, in the UN Comtrade (2016) data, sand trade varied between different past years by a factor of 3.5 (cf. figure D1). Second, based on several estimates (Foreign Policy, 2010; The Asia Miner,

[3] We increase the labor input into the USA's transport sector such that its output and input values equal each other. The USA has been chosen because it is the world's largest economy. As a result of the data adjustment, the output of the USA's transport sector increases by less than one per mill, making the assumption innocuous.

2014; FAZ, 2016), Singapore's planned future sand reclamation projects will increase its sand demand by a factor of between 1.7 and 6.8.

4.4 Historical policies

Historically, the $SAND$ sector has been subject to various policies, particularly export bans in Vietnam and Malaysia. To eliminate these export bans, we recalibrate the model such that all trade barriers to $SAND$ trade are zero. Although we can set the export tariffs to zero, the effect of the export bans must be estimated. To this end, we apply ordinary least squares (OLS) to the econometric model in (1) to quantify the impact of export bans.

$$\log\left(\frac{\pi_{SAND,r,s}}{\pi_{SAND,s,s}}\right) = E_r - E_s - \theta_i\left(\log\tau^t_{SAND,r,s} + \log\tilde{\delta}_{SAND,r,s}\right) + \varepsilon_{r,s} \tag{1}$$

$\pi_{SAND,r,s}$ denotes the trade share, the fraction of $SAND$ that region r exports to region s. E_r and E_s are exporter and importer fixed effects that represent a combination of production costs and productivity in the $SAND$ sector of regions r and s, respectively. $\tau^t_{SAND,r,s}$ captures the observable trade costs that include tariffs and transport costs. The iceberg trade costs, which depend on whether there is a ban in force on exports from region r to s, are denoted by $\tilde{\delta}_{SAND,r,s}$. They are approximated by equation (2), which is plugged into equation (1). $\varepsilon_{r,s}$ is an idiosyncratic error term.

$$\log\tilde{\delta}_{SAND,r,s} = \mu\log dist_{r,s} + \beta ban_{r,s} \tag{2}$$

$dist_{r,s}$ represents the geographical distance between the regions r and s, and μ is the elasticity of iceberg trade costs with respect to this distance. The dummy variable $ban_{r,s}$ equals one if there is a ban in force on exports from r to s. The coefficient β quantifies the impact of this ban on iceberg trade costs. We only observe very few trade flows for $SAND$; therefore, we do not include other dummies in equation (2).

4.5 Examined policies

In the scenario simulations, a uniform sand-specific tax on top of the market price of sand internalizes the social (environmental) damages of sand extraction. This sand tax is implemented differently in several counterfactual policy scenarios; it is denoted by $\tau^{S'}$ and measured in US$ per ton. It can be implemented as an exogenous tax or emerge as an endogenous market outcome of a Sand Extraction Certificate Trading System (SEATS)

with a given limit of sand extraction.

We assume that the marginal social damages created by sand extraction are proportional to the amount of sand (and gravel) extracted in r, labeled S_r and measured in tons. Let $X_{SAND,r}$ denote the monetary value in the baseline scenario of sand sales measured in US$. Then, a sand intensity $\frac{S_r}{X_{SAND,r}}$ can be defined for the baseline to characterize the amount of sand extracted per monetary unit of sand sold, measured in tons per US$. This quantity differs across regions r but is assumed to stay constant across the scenarios for each r. Thus, if, for example, the output $Q_{SAND,r}$ of the sand sector in r measured in real currency units increases by one percent in a counterfactual scenario, S_r will rise by one percent as well. This assumption is required for the distinct policy implementations.

In the policy analysis, we investigate the effects of three types of sand taxes: an export tax (export tariff in scenario ExpTax) imposed on the sand sector of all sand-exporting regions, an import tax (import tariff in scenario ImpTax) levied in Singapore and an output (sales) tax (in scenario OutTax) on all deliveries (total extraction) of sand.

Depending on the scenario, we convert the sand tax into an ad valorem export, import or output (sales) tax (or, respectively, tariff) to ease the implementation in the model and to mimic the implementation of real-world policies. In this conversion, we consider the existence of regional differences in sand intensities.

To illustrate this conversion, we derive Singapore's import tax. Similar to the implementation procedure of border carbon adjustments, the sand tax $\tau^{S'}$ per unit of sand is multiplied by the exporter-specific sand intensity and divided by the counterfactual sand price measured relative to the baseline to eliminate monetary effects. This procedure yields a dimensionless exporter-specific ad valorem import tariff $\tau^{m'}_{SAND,r,s}$ required for the counterfactual policy scenario, which reflects the physical sand content of sand trade from r to s measured in pecuniary terms.

The corresponding transformations result in the ad valorem export tax rate ($\tau^{e'}_{SAND,r,s}$) and the ad valorem output (sales) tax rate ($\tau^{o'}_{SAND,r}$). Whereas the revenues from the import tax are distributed to Singapore's (SGP's) representative consumer as a lump sum, the revenues from the other taxes are redistributed to the consumer of the corresponding sand-extracting region r. While the output tax affects the total sand sales (extraction), the export tax affects the exported fraction only.

5 Mathematical formulation

5.1 Approach

The following sections express the model in mathematical terms. The underlying general equilibrium model is formulated as an MCP (Mixed Complementarity Problem), programmed in GAMS (General Algebraic Modeling System; Bussieck and Meeraus, 2004) and solved by using the PATH algorithm (Dirkse and Ferris, 1995). The trade model setup follows the implementation of the theory of Eaton and Kortum (2002) by Caliendo and Parro (2015) and Pothen and Hübler (2018). This section details the model consisting of equations derived from zero-profit conditions or the theory of Eaton and Kortum (2002) (subsections 5.2 to 5.5), market clearing conditions (subsection 5.6), the income balance condition (subsection 5.7) and policy-related constraints (subsection 5.8).

The model equations are written in terms of relative changes. They characterize a counterfactual (scenario) value relative to the baseline value normalized to unity, e.g., 1.1 in the counterfactual scenario compared to 1.0 in the baseline implies a 10% increase in the variable under consideration. In the literature based on Eaton and Kortum (2002), this formulation is known as "exact hat algebra" (Dekle et al., 2008). A comparable approach in the literature based on computable general equilibrium (CGE) models is the "calibrated share form" of CES functions (Böhringer et al., 2003). Regarding the formulation in terms of changes, the model differs from that of Pothen and Hübler (2018). The formulation in terms of changes has the advantage that no structural estimation is required for model calibration.

We employ the following notation. For a model variable or parameter "x", x denotes the baseline value that is normally given by the benchmark data of the model calibration. x' denotes the corresponding value in the counterfactual simulation, and $\hat{x} = \frac{x'}{x}$ is the change between the counterfactual and the baseline, which will be applied in the following analysis. In particular, we quantify the economic effects of changing the sand tax from a baseline value of $\tau^S = 0$ to a counterfactual value of $\tau^{S'} > 0$.

5.2 Consumption

5.2.1 Cost functions

Referring to figure A1, this subsection defines the cost functions of the demand side. Equation (3) describes the change in the true-cost-of-living index between the counterfactual scenario and the baseline, denoted by $\hat{c}_s^C$. It is derived from the CES utility function with

the elasticity of substitution σ^C. The variable $\hat{c}_s^{CE}$ denotes the change in the cost index of the energy aggregate, while $\hat{c}_s^{CN}$ denotes the change in the costs of the non-energy aggregate. β_s^C is the value share of the energy aggregate in the baseline.

$$\hat{c}_s^C = \left(\beta_s^C(\hat{c}_s^{CE})^{1-\sigma^C} + (1-\beta_s^C)(\hat{c}_s^{CN})^{1-\sigma^C}\right)^{\frac{1}{1-\sigma^C}} \tag{3}$$

The change $\hat{c}_s^{CE}$ in the cost index of the energy aggregate in consumption is computed similarly. It depends on the change in the price of good i ($\hat{P}_{i,s}$), the value share of this good in the energy aggregate ($\beta_{i,s}^{CE}$) and the elasticity of substitution between these goods (σ^{CE}). Any consumption tax ($\tau_{i,s}^c$) does not appear in equation (4) because the tax rate does not change between the baseline and the counterfactual scenario. We use the simplified notation $[CE]$ to symbolize the subset of energy sectors $[CE] = \{COAL, NGAS, PETR, CRUD, ELEC\}$ in the summation.

$$\hat{c}_s^{CE} = \left(\sum_{i|i\in[CE]} \beta_{i,s}^{CE}(\hat{P}_{i,s})^{1-\sigma^{CE}}\right)^{\frac{1}{1-\sigma^{CE}}} \tag{4}$$

The change in the cost index of the non-energy aggregate in consumption ($\hat{c}_s^{CN}$) is computed analogously.

$$\hat{c}_s^{CN} = \left(\sum_{i|i\in[CN]} \beta_{i,s}^{CN}(\hat{P}_{i,s})^{1-\sigma^{CN}}\right)^{\frac{1}{1-\sigma^{CN}}} \tag{5}$$

5.2.2 Demand functions

This subsection explains the demand functions. They describe the change in the representative consumer's demand for good i. To simplify the exposition, we split the complex demand functions derived from the CES utility function into per-unit demand functions for each nest. The function $\hat{d}_s^{C,CE}$, for instance, describes the change in demand for the non-energy aggregate per consumption unit:

$$\hat{d}_s^{C,CE} = \left(\frac{\hat{c}_s^C}{\hat{c}_s^{CE}}\right)^{\sigma^C} \tag{6}$$

The change in demand for the non-energy aggregate $\hat{d}_s^{C,CN}$ can be written analogously:

$$\hat{d}_s^{C,CN} = \left(\frac{\hat{c}_s^C}{\hat{c}_s^{CN}}\right)^{\sigma^C} \tag{7}$$

The expression $\hat{d}_{i,s}^{CE,i}$ represents the change in the demand for good $i \in [CE]$ per consumption unit of the energy aggregate. Again, any consumption tax $\tau_{i,s}^{c}$ does not appear in the demand function because it does not change between the baseline and the counterfactual scenario.

$$\hat{d}_{i,s}^{CE,i} = \left(\frac{\hat{c}_s^{CE}}{\hat{P}_{i,s}} \right)^{\sigma^{CE}} \quad \forall i \in [CE] \tag{8}$$

The demand for the non-energy good $i \in [CN]$ per consumption unit of the non-energy aggregate is expressed as

$$\hat{d}_{i,s}^{CN,i} = \left(\frac{\hat{c}_s^{CN}}{\hat{P}_{i,s}} \right)^{\sigma^{CN}} \quad \forall i \in [CN] \tag{9}$$

The combination of equations (6) to (9) yields the following expression for the change in consumption of good i distinguishing between energy goods and non-energy goods:

$$\hat{C}_{i,s} = \begin{cases} \frac{\hat{Y}_s}{\hat{c}_s^C} \cdot \hat{d}_s^{C,CE} \cdot \hat{d}_{i,s}^{CE,i} & \text{if } i \in [CE] \\ \frac{\hat{Y}_s}{\hat{c}_s^C} \cdot \hat{d}_s^{C,CN} \cdot \hat{d}_{i,s}^{CN,i} & \text{if } i \in [CN] \end{cases} \tag{10}$$

5.3 Production

5.3.1 Cost functions

Referring to figure A2, this subsection defines the cost functions of the production side. To this end, the per-unit cost function ($c_{i,r}$) is split into per-unit cost functions for each nest of the production technology depicted by figure A2. The variable $\hat{c}_{i,s}^{KL}$, for instance, represents the change in the Cobb-Douglas cost index of value added in the production of good i in region r. The parameter $\beta_{i,r}^{KL}$ represents the value share of capital in the $KL_{i,r}$ nest.

$$\hat{c}_{i,r}^{KL} = (\hat{P}_r^K)^{\beta_{i,r}^{KL}} \cdot (\hat{P}_r^L)^{1-\beta_{i,r}^{KL}} \tag{11}$$

Equation (12) describes the change in the cost index of the fossil fuel nest $FF_{i,r}$ of i in r, $\hat{c}_{i,r}^{FF}$. $\beta_{j,i,r}^{FF}$ denotes the value share of fossil fuel j in the FF nest. Let $[FF]$ symbolize the subset of all fossil fuel sectors $[FF] = \{COAL, NGAS, PETR, CRUD\}$ so that

$$\hat{c}_{i,r}^{FF} = \prod_{j \in [FF]} (\hat{P}_{j,r})^{\beta_{j,i,r}^{FF}} \tag{12}$$

$\hat{c}^{E}_{i,r}$ characterizes the change in the cost index of the energy nest $E_{i,r}$. The value share of fossil fuels is denoted $\beta^{E}_{i,r}$. σ^{E} symbolizes the nonunitary elasticity of substitution, and $\hat{P}_{ELEC,r}$ is the change in the price of electricity $ELEC$.

$$\hat{c}^{E}_{i,r} = \left(\beta^{E}_{i,r}(\hat{c}^{FF}_{i,r})^{1-\sigma^{E}} + (1-\beta^{E}_{i,r})(\hat{P}_{ELEC,r})^{1-\sigma^{E}}\right)^{\frac{1}{1-\sigma^{E}}} \tag{13}$$

The $KLE_{i,r}$ nest combines the value added and the energy aggregates with the elasticity of substitution σ^{KLE}. The change in its per-unit cost index is denoted by $\hat{c}^{KLE}_{i,r}$. $\beta^{KLE}_{i,r}$ is the value share of value added in the baseline.

$$\hat{c}^{KLE}_{i,r} = \left(\beta^{KLE}_{i,r}(\hat{c}^{KL}_{i,r})^{1-\sigma^{KLE}} + (1-\beta^{KLE}_{i,r})(\hat{c}^{E}_{i,r})^{1-\sigma^{KLE}}\right)^{\frac{1}{1-\sigma^{KLE}}} \tag{14}$$

Non-energy intermediate good inputs are combined in the $Z_{i,r}$ nest by using a Leontief function. Thus, the change in the corresponding price index $\hat{c}^{Z}_{i,r}$ is a weighted average of their prices. The weights are given by the corresponding value shares $\beta^{Z}_{j,i,r}$. $[Z]$ symbolizes the set of all non-energy (intermediate) goods, i.e., all goods except energy goods and the investment good $INVS$.

$$\hat{c}^{Z}_{i,r} = \sum_{j\in[Z]} \beta^{Z}_{j,i,r}\hat{P}_{j,r} \tag{15}$$

The change in the per-unit input costs $\hat{c}_{i,r}$ is expressed as equation (16), where $\beta^{KLEM}_{i,r}$ is the value share of the $KLE_{i,r}$ aggregate, and σ^{KLEM} is the elasticity of substitution.

$$\hat{c}_{i,r} = \left(\beta^{KLEM}_{i,r}(\hat{c}^{KLE}_{i,r})^{1-\sigma^{KLEM}} + (1-\beta^{KLEM}_{i,r})(\hat{c}^{Z}_{i,r})^{1-\sigma^{KLEM}}\right)^{\frac{1}{1-\sigma^{KLEM}}} \tag{16}$$

5.3.2 Demand functions

The demand for intermediate inputs and primary factors is split into several per-unit demand functions. The change in the demand for capital within the value added nest, for instance, is denoted by $\hat{d}^{KL,K}_{i,r}$ and depends on the relationship between the changes in the cost index of the value added aggregate $\hat{c}^{KL}_{i,r}$ and the rental rate of capital $\hat{P}^{K}_{r}$.

$$\hat{d}^{KL,K}_{i,r} = \frac{\hat{c}^{KL}_{i,r}}{\hat{P}^{K}_{r}} \tag{17}$$

Demand for labor by the $KL_{i,r}$ nest $\hat{d}_{i,r}^{KL,L}$ can be expressed analogously.

$$\hat{d}_{i,r}^{KL,L} = \frac{\hat{c}_{i,r}^{KL}}{\hat{P}_r^L} \tag{18}$$

The change in the demand for fossil fuel j by the fossil fuel nest, $\hat{d}_{j,i,r}^{FF,i}$, is also derived from a Cobb-Douglas function.

$$\hat{d}_{j,i,r}^{FF,i} = \frac{\hat{c}_{i,r}^{FF}}{\hat{P}_{j,r}} \ \forall j \in [FF] \tag{19}$$

Likewise, the change in demand for the fossil fuel aggregate by the energy aggregator is defined as

$$\hat{d}_{i,r}^{E,FF} = \left(\frac{\hat{c}_{i,r}^{E}}{\hat{c}_{i,r}^{FF}} \right)^{\sigma^E} \tag{20}$$

The following equation defines the change in the demand for electricity by the energy aggregator:

$$\hat{d}_{i,r}^{E,ELEC} = \left(\frac{\hat{c}_{i,r}^{E}}{\hat{P}_{ELEC,r}} \right)^{\sigma^E} \tag{21}$$

Correspondingly, the change in the demand for value added in the $KLE_{i,r}$ nest reads

$$\hat{d}_{i,r}^{KLE,KL} = \left(\frac{\hat{c}_{i,r}^{KLE}}{\hat{c}_{i,r}^{KL}} \right)^{\sigma^{KLE}} \tag{22}$$

and the change in the demand for the energy in the $KLE_{i,r}$ nest reads

$$\hat{d}_{i,r}^{KLE,E} = \left(\frac{\hat{c}_{i,r}^{KLE}}{\hat{c}_{i,r}^{E}} \right)^{\sigma^{KLE}} \tag{23}$$

The demand for good j (including $SAND$) by the aggregator of non-energy intermediates is derived from a Leontief function and thus remains unchanged in the counterfactual scenario.

$$\hat{d}_{j,i,r}^{Z,j} = 1 \ \forall j \in [Z] \tag{24}$$

Equations (25) and (26) show the changes in the demand for the $KLE_{i,r}$ aggregate ($\hat{d}_{i,r}^{KLEM,KLE}$) and the non-energy intermediate aggregate ($\hat{d}_{i,r}^{KLEM,Z}$), respectively.

$$\hat{d}_{i,r}^{KLEM,KLE} = \left(\frac{\hat{c}_{i,r}^{KLEM}}{\hat{c}_{i,r}^{KLE}}\right)^{\sigma^{KLEM}} \tag{25}$$

$$\hat{d}_{i,r}^{KLEM,Z} = \left(\frac{\hat{c}_{i,r}^{KLEM}}{\hat{c}_{i,r}^{Z}}\right)^{\sigma^{KLEM}} \tag{26}$$

Equation (27) defines the change in demand for intermediate input j by sector i in r, $\hat{Z}_{j,i,r}$. Here, we distinguish between three types of goods: fossil fuels $[FF]$, electricity $ELEC$, and non-energy intermediates $[Z]$ including $SAND$.

$$\hat{Z}_{j,i,r} = \begin{cases} \frac{\hat{x}_{i,r}}{\hat{c}_{i,r}} \cdot \hat{d}_{i,r}^{KLEM,KLE} \cdot \hat{d}_{i,r}^{KLE,E} \cdot \hat{d}_{i,r}^{E,FF} \cdot \hat{d}_{j,i,r}^{FF,i} & \text{if } j \in [FF] \\ \frac{\hat{x}_{i,r}}{\hat{c}_{i,r}} \cdot \hat{d}_{i,r}^{KLEM,KLE} \cdot \hat{d}_{i,r}^{KLE,E} \cdot \hat{d}_{i,r}^{E,ELEC} & \text{if } j = ELEC \\ \frac{\hat{x}_{i,r}}{\hat{c}_{i,r}} \cdot \hat{d}_{i,r}^{KLEM,Z} \cdot \hat{d}_{j,i,r}^{Z,j} & \text{if } j \in [Z] \end{cases} \tag{27}$$

Sector i's change in the demand for capital is expressed as follows:

$$\hat{K}_{i,r} = \frac{\hat{x}_{i,r}}{\hat{c}_{i,r}} \cdot \hat{d}_{i,r}^{KLEM,KLE} \cdot \hat{d}_{i,r}^{KLE,KL} \cdot \hat{d}_{i,r}^{KL,K} \tag{28}$$

Likewise, sector i's change in the demand for labor reads

$$\hat{L}_{i,r} = \frac{\hat{x}_{i,r}}{\hat{c}_{i,r}} \cdot \hat{d}_{i,r}^{KLEM,KLE} \cdot \hat{d}_{i,r}^{KLE,KL} \cdot \hat{d}_{i,r}^{KL,L} \tag{29}$$

5.4 Trade

This subsection considers international trade based on the theory of Eaton and Kortum (2002) and the implementations by Caliendo and Parro (2015) and Pothen and Hübler (2018). Equation (30) represents the change in the price index of sector i in region s, $\hat{P}_{i,s}$, between the baseline and the counterfactual scenario; it depends on the changes in per-unit costs ($\hat{c}_{i,r}$) and observable trade costs ($\hat{\tau}_{i,r,s}^{t}$). The baseline trade share ($\pi_{i,r,s}$) indicates the importance of changes in per-unit input cost or trade cost changes in region r for the price in region s. If region r is an important supplier of region s in the baseline, an increase in input or trade costs will have a large effect on s's price index in the counterfactual scenario. The absolute productivity ($\hat{T}_{i,r}$) represents a sector's efficiency of converting the

input bundle into the output. It does not, however, appear in equation (30) because it does not change between the baseline and the counterfactual scenario ($\hat{T}_{i,r} = 1$).

$$\hat{P}_{i,s} = \sum_r \pi_{i,r,s} \left(\hat{c}_{i,r} \cdot \hat{\tau}^t_{i,r,s}\right)^{-\theta_i} \tag{30}$$

Let $\pi'_{i,r,s}$ denote the trade share, i.e., the fraction of good i that s purchases from r, in the counterfactual scenario. $\pi'_{i,r,s}$ can be written as a function that increases with the price index ($\hat{P}_{i,s}$) and decreases with the per-unit production costs of i in r ($\hat{c}_{i,r}$) multiplied by the (observable) trade costs of shipping good i from r to s ($\hat{\tau}^t_{i,r,s}$), where the arguments are measured in terms of changes:

$$\pi'_{i,r,s} = \pi_{i,r,s} \left(\frac{\hat{P}_{i,s}}{\hat{c}_{i,r} \cdot \hat{\tau}^t_{i,r,s}} \right)^{\theta_i} \tag{31}$$

Similarly, the change in the observable trade costs ($\hat{\tau}^t_{i,r,s}$) is driven by the endogenous changes in transport costs and, in the case of $SAND$, the tax or tariff under examination.

$$\hat{\tau}^t_{i,r,s} = \frac{(1+\tau^{m'}_{i,r,s})(1+\psi_{i,r,s}P^{ITR'})(1-\tau^{e'}_{i,r,s}+\tau^{o'}_{i,r})}{(1+\tau^{m}_{i,r,s})(1+\psi_{i,r,s}P^{ITR})(1-\tau^{e}_{i,r,s}+\tau^{o}_{i,r})} \tag{32}$$

The observable trade costs consist of four components. The first is the import tariff ($\tau^m_{i,r,s}$), which can change in the case of $SAND$ but remains constant in other sectors. The second are the transport costs, which, in turn, consist of the constant input of international transport services per unit of good i shipped from r to s ($\psi_{i,r,s}$) and the endogenous price of international transport services (P^{ITR}). The third is the export tariff $\tau^e_{i,r,s}$, which can also change in the case of $SAND$. The fourth is the output tax on $SAND$ ($\tau^o_{i,r}$), which equals zero in the baseline.

5.5 Transportation

International transport services are assumed to be a global Cobb-Douglas aggregate of inputs from transport sectors in all regions r. The change in their price $\hat{P}^{ITR}$ hence depends only on price changes of regional transport services, $\hat{P}_{TRNS,r}$, and the corresponding value shares ζ_r.

$$\hat{P}^{ITR} = \prod_r (\hat{P}_{TRNS,r})^{\zeta_r} \tag{33}$$

5.6 Markets

5.6.1 Transportation market clearing

Referring to the previous subsection, the following equation represents the market clearing condition for international (global) transportation services in the counterfactual scenario, where $Q^{ITR'}$ denotes the supply of international transport services.

$$Q^{ITR'} = \sum_{i,r,s} \psi_{i,r,s} \frac{(1 - \tau^{e'}_{i,r,s} + \tau^{s'}_{i,r,s})}{\tau^{t'}_{i,r,s}} \pi^{'}_{i,r,s} D^{'}_{i,s} \tag{34}$$

5.6.2 Goods market clearing

Market clearance is required in all production sectors i (including $SAND$ and $INVS$). For this purpose, let us write the counterfactual sales of sector i in region r ($X'_{i,r}$) as a positive function of the expenditures on good i in all regions s ($D^{'}_{i,s}$), the fraction of these expenditures purchased from r ($\pi^{'}_{i,r,s}$) and a negative function of the (observable) trade costs ($\tau^{t'}_{i,r,s}$) between r and s. In the transportation sector $TRNS$, the sales to the international transport services ($\zeta_r Q^{ITR'} P^{ITR'}$) are added to the right-hand side of the following equation.

$$X'_{i,r} = \sum_{s} \pi^{'}_{i,r,s} \frac{D^{'}_{i,s}}{\tau^{t'}_{i,r,s}} \tag{35}$$

Furthermore, the expenditures on good i in region s must equal the sum of the expenditures on consumption ($C'_{i,s}$) and intermediate good inputs ($Z'_{i,j,s}$):

$$D'_{i,s} = P'_{i,s} \left(C'_{i,s} + \sum_{j} Z'_{i,j,s} \right) \tag{36}$$

5.6.3 Factor market clearing

A well-defined model solution requires clearance of all factor markets as well. The following capital market clearing condition equates the region-specific, exogenous and constant capital endowment ($\bar{K}_r$) with the endogenous counterfactual demand for capital ($K'_{i,r}$) by all sectors i in region r. This equilibrium condition determines the rental rate of capital ($P^{K'}_r$), where capital includes natural resources.

$$\bar{K}_r = \sum_{i} K'_{i,r} \tag{37}$$

Finally, the wage rate ($P^{L'}_r$) is determined by the corresponding labor market clearing

condition:

$$\bar{L}_r = \sum_i L'_{i,r} \tag{38}$$

5.7 Income

The income (value) of the representative consumers of region s in the counterfactual scenario (Y'_s) consists of capital income ($P_s^{K'} \bar{K}_s$), labor income ($P_s^{L'} \bar{L}_s$), redistributed tax revenues (Ξ'_s) and the current account deficit (Δ_s).

$$Y'_s = P_s^{K'} \bar{K}_s + P_s^{L'} \bar{L}_s + \Xi'_s + \Delta_s \tag{39}$$

This income balance condition must hold in each model equilibrium. Whereas Δ_s remains unchanged across scenarios, the values of the other income sources change endogenously.

The corresponding income value in the baseline (Y_s) is given so that the income change $\hat{Y}_s$ can be derived. Based on that, the welfare change between the counterfactual scenario and the baseline can be expressed as

$$\hat{w}_s = \frac{\hat{Y}_s}{\hat{c}_r^C} \tag{40}$$

where $\hat{c}_r^C$ denotes the change in the true-cost-of-living index, i.e., the price of the optimal consumption bundle derived from the CES utility function in figure A1.

5.8 Policies

This subsection rephrases the policies discussed in section 4.5 in a mathematical form. All changes in the model solution are driven by adding a positive sand tax to the price of sand (and gravel) $\tau^{S'}$ in the counterfactual scenario. Depending on the policy scenario, this tax is imposed on imports (to Singapore), exports (of the Southeast Asian suppliers) or total output (total sales of the Southeast Asian suppliers) of *SAND*. The corresponding ad valorem tax (tariff) rates are derived as explained in section 4.5.

Equation (41) expresses Singapore's ad-valorem import tariff on sand in the counterfactual scenario, $\tau^{m'}_{SAND,r,s}$. The division of $\tau^{S'}$ by the counterfactual sand price measured relative to the baseline price ($\hat{P}_{SAND,r}$) eliminates monetary price effects. S_r denotes the amount of sand (and gravel) extracted in r; $X_{SAND,r}$ is the monetary value of sand sales; and $\frac{S_r}{X_{SAND,r}}$ is the resulting sand intensity that is constant across scenarios. The tariff revenues accrue to the representative consumer of the importing region s, i.e., Singapore

(SGP), as a lump sum.

$$\tau^{m'}_{SAND,r,s} = \begin{cases} \frac{\tau^{S'}}{\hat{P}_{SAND,r}} \cdot \frac{S_r}{X_{SAND,r}} & \text{if } r \neq s \ \wedge \ s = SGP \\ \textit{not applicable} & \text{otherwise} \end{cases} \quad (41)$$

The export tariff in the counter-factual scenario ($-\tau^{e'}_{SAND,r,s}$) is computed similarly. The minus sign is necessary because, following the GTAP approach, we implement export subsidies rather than export tariffs. Notably, the revenues from export tariffs are redistributed to the representative consumer of the exporting region $r \in [SX]$ as a lump sum, where $[SX]$ symbolizes the subset of sand exporters $\{KHM, MMR, MYS, PHL, VNM\}$.

$$-\tau^{e'}_{SAND,r,s} = \begin{cases} \frac{\tau^{S'}}{\hat{P}_{SAND,r}} \cdot \frac{S_r}{X_{SAND,r}} & \text{if } r \neq s \wedge \ r \in [SX] \\ \textit{not applicable} & \text{otherwise} \end{cases} \quad (42)$$

The (output) sales tax on sand in the counter-factual scenario ($\tau^{o'}_{SAND,r}$) is computed accordingly.

$$\tau^{o'}_{SAND,r} = \begin{cases} \frac{\tau^{S'}}{\hat{P}_{SAND,r}} \cdot \frac{S_r}{X_{SAND,r}} & \text{if } \ r \in [SX] \\ \textit{not applicable} & \text{otherwise} \end{cases} \quad (43)$$

Unlike the tariff imposed on exports, it is levied on all sales of sand including those to domestic consumers and firms; i.e., the tax base is broader. Revenues are redistributed to the representative consumer of the sand-extracting country r as a lump sum.

These policy definitions complete the model description.

References

Aguiar, A., Narayanan, B., and McDougall., R. (2016). An Overview of the GTAP 9 Data Base. *Journal of Global Economic Analysis*, 1(1):181–208.

The Asia Miner (2014). Vietnam – Vanguard in sand supply agreement. Camberwell, Australia, `http://www.asiaminer.com/news/latest-news/5971-vietnam-vanguard-in-sand-supply-agreement.html#.VRlMdeE6_Kg` (accessed 06/2018).

Böhringer, C., Rutherford, T. F., and Wiegard, W. (2003). Computable General Equilibrium Analysis: Opening a Black Box. *ZEW Discussion Paper*, 03-56.

Bussieck, M. R. and Meeraus, A. (2004). General Algebraic Modeling System (GAMS). In Kallrath, J., editor, *Modeling Languages in Mathematical Optimization*, 137–157. Springer US, Boston, MA, USA.

Caliendo, L. and Parro, F. (2015). Estimates of the Trade and Welfare Effects of NAFTA. *Review of Economic Studies*, 82(1):1–44.

Dekle, R., Eaton, J., and Kortum, S. (2008). Global Rebalancing with Gravity: Measuring the Burden of Adjustment. *IMF Staff Papers*, 55(3):511–540.

Dirkse, S. P. and Ferris, M. C. (1995). The PATH Solver: A Non-Monotone Stabilization Scheme for Mixed Complementarity Problems. *Optimization Methods and Software*, 5(2):123–156.

Eaton, J. and Kortum, S. (2002). Technology, Geography, and Trade. *Econometrica*, 70(5):1741–1779.

FAZ (2016). Auf den Spuren der Sandfresser. Frankfurter Allgemeine, Frankfurt, Germany, `http://www.faz.net/aktuell/gesellschaft/rohstoffabbau-in-suedostasien-sand-verschwindet-14113235.html` (accessed 06/2018)

Foreign Policy (2010). The Sand Smugglers. `http://foreignpolicy.com/2010/08/04/the-sand-smugglers/` (accessed 06/2018).

Lutter, S., Lieber, M., and Giljum., S. (2015). Global Material Flow Database. Material Extraction Data. Technical Report, Version 2015.1. `http://www.materialflows.net/fileadmin/docs/materialflows.net/WU_MFA_Technical_report_2015.1_final.pdf`

OECD (2018). Export restrictions on industrial raw materials, Paris, France, `https://qdd.oecd.org/subject.aspx?Subject=ExportRestrictions_IndustrialRawMaterials` (accessed 03/2018).

Paltsev, S., Reilly, J. M., Jacoby, H. D., Eckaus, R. S., McFarland, J., Sarofim, M., and Babiker, M. A. M. (2005). The MIT Emissions Prediction and Policy Analysis (EPPA) Model: Version 4. *Joint Program on the Science and Policy of Global Change Reports*, 125.

Pothen, F. and Hübler, M. (2018). The Interaction of Climate and Trade Policy. *European Economic Review*, 107:1–26.

Statista (2018). *Average U.S. price of sand and gravel 2007–2017*. The Statistics Portal, Hamburg, Germany, London, UK, New York, USA. `www.statista.com/statistics/219381/sand-and-gravel-prices-in-the-us/` (accessed 05/2018).

UN Comtrade (2016). *United Nations Trade Statistics*. New York, USA. `https://comtrade.un.org/` (accessed 10/2016).

Van der Werf, E. (2008). Production functions for climate policy modeling: An empirical analysis. *Energy Economics*, 30(6):2964–2979.
